# Porcine Circovirus Type 2 ORF5 Protein Induces Autophagy to Promote Viral Replication via the PERK-eIF2α-ATF4 and mTOR-ERK1/2-AMPK Signaling Pathways in PK-15 Cells

**DOI:** 10.3389/fmicb.2020.00320

**Published:** 2020-02-28

**Authors:** Jiangman Lv, Yanfen Jiang, Quanwen Feng, Zhixin Fan, Ying Sun, Panpan Xu, Yufeng Hou, Xiuping Zhang, Yuxin Fan, Xingang Xu, Yanming Zhang, Kangkang Guo

**Affiliations:** ^1^College of Veterinary Medicine, Northwest A&F University, Yangling, China; ^2^College of Animal Science, Tarim University, Alar, China

**Keywords:** autophagy, ORF5 protein, PK-15 Cell, *Porcine circovirus* 2, virus replication

## Abstract

Porcine circovirus type 2 (PCV2) is the primary causative agent that causing porcine circovirus-associated disease (PCVAD). The open reading frame 5 (ORF5) protein is a newly discovered non-structural protein in PCV2, which the function in viral pathogenesis remains unknown. The aim of this study was to investigate the mechanism of PCV2 ORF5 protein on autophagy and viral replication. The pEGFP-tagged ORF5 gene was ectopic expressed in PK-15 cells and an ORF5-deficient PCV2 mutant strain (PCV2^ΔORF5^) were used to infected PK-15 cells. This study demonstrated that the ORF5 is essential for the of PCV2-induced autophagy. The ORF5 protein triggers the phosphorylation of PERK, eIF2α and the expression of downstream transcription factor ATF4. In addition, ORF5 protein activated the AMPK-ERK1/2-mTOR signaling pathways. These findings suggest that ORF5 play essential roles in the induction of autophagy by PCV2. We further revealed that PCV2 ORF5 promotes viral replication through PERK-eIF2α-ATF4 and AMPK-ERK1/2-mTOR pathways. In conclusion, we showed that PCV2 ORF5 induces autophagy to promote virus replication in PK-15 cells.

## Graphical Abstract

PCV2 ORF5 inducing autophagy to promote PCV2 replication.

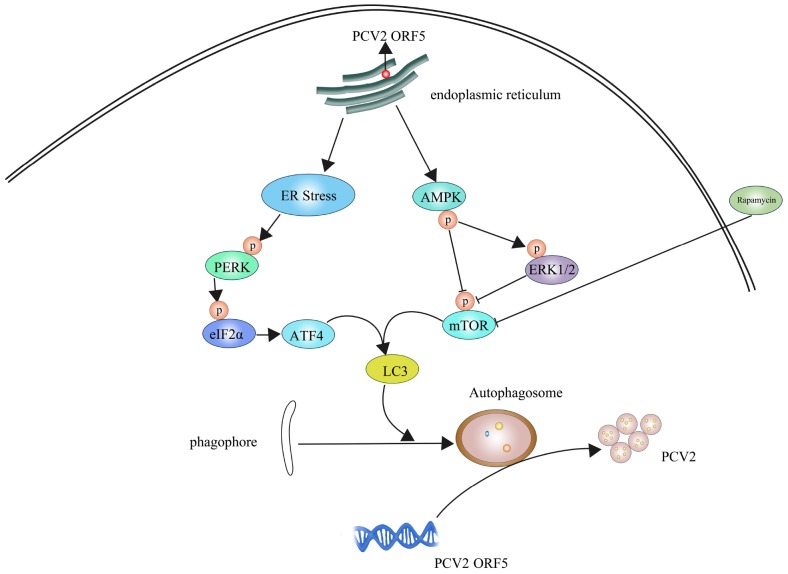

## Introduction

Porcine circovirus (PCV) is the smallest virus found to infect mammals so far (Delwart and Li, 2012). Its genome is approximately 1.7 kb in length, and is a covalently closed single strand negative circular DNA (Meng, 2012). PCV2 is the main pathogen of porcine circovirus-associated disease (PCVAD) causing huge economic losses to the pig industry (Rosell et al., 2000; Wilfred et al., 2018). PCV2 infection is widespread in almost all pig-raising countries and became an important factor affecting the pig industry. Autophagy can be involved in the replication process of many viruses (Jackson, 2015). PCV2 can induce cellular autophagy in host cells such as PK-15 cells, and the cellular autophagy promotes viral replication (Eng et al., 2016). PCV2 level is significantly reduced after interfering with autophagy process (Zhu et al., 2012a, b) while PCV2 replication enhanced by activating the PERK (RNA-activated protein kinase-like endoplasmic reticulum kinase)-eIF2α (eukaryotic initiation factor 2α)-ATF4 (activating transcription factor 4) axis (Zhou et al., 2016), indicated the pathogenesis of PCV2 may be related to autophagy.

Autophagy is a physiological activity in which organisms rely on lysosomes to self-degrade their own macromolecular proteins and damaged organelles (Zhao et al., 2018). However, the mechanism of how autophagy involved in PCV2 replication is still unclear. The ORF5 protein, a newly identified protein encoded by PCV2 has been demonstrated located to the endoplasmic reticulum and induces endoplasmic reticulum stress (ERS) in host cells (Lv et al., 2015). Upon the viral infection, unfolded proteins accumulate in the endoplasmic reticulum and trigger the unfolded protein reaction (UPR) and eventually leads to ERS (Rabinowitz and White, 2010). During hepatitis C virus (HCV) infection, the silence of PERK signaling pathway in the UPR led to decreased autophagy and the titer of the virus also decreased (Wang et al., 2014). In addition, Bluetongue virus (BTV) infection activates the PERK/eIF2α pathway to mediate autophagy and promote viral replication (Wang et al., 2009).

Furthermore, the mTOR (mammalian target of rapamycin) pathway also plays a key role in regulating autophagy (Hay and Sonenberg, 2004). After mammalian synthesis of LC3 (autophagy marker light chain 3), a small stretch of peptide chain at the end of LC3I is cleaved to form LC3II under the catalytic shear of Atg3, Atg4, and Atg7, and this process is a key step in the extension of autophagosome membrane (Fujita et al., 2008). In mammalian cells, adenosine monophosphates-activated protein kinase (AMPK) negatively regulates mTOR by activating TSC2 (tuberous sclerosis complex-2) protein (Madeo et al., 2015). Also it is reported that I, III phosphatidylinositol 3-kinase (PI3K), mitogen-activated protein kinase (MAPK), reactive oxygen species (ROS)/c-Jun N-terminal kinase (JNK) and AMPK are involved in the formation of autophagosomes (Zhou et al., 2015; Zhong et al., 2017). In this study, we aimed to investigated the effect of PCV2 ORF5 protein on autophagy. We found that ORF5 play important roles in PCV2-activated autophagy. Remarkably, we found that ORF5 facilitate viral replication through PERK-eIF2α-ATF4 and AMPK-ERK1/2-mTOR pathways.

## Materials and Methods

### Cell Line

Porcine kidney-derived cell line PK-15 cells (ATCC: CCL-33) were maintained in Dulbecco’s Modified Eagle’s Medium (DMEM, Hyclone, United States), supplemented with 10% Fetal bovine serum (FBS) (ZATA LIFE, United States), 1% L-glutamine, 1% non-essential amino acids, 100 units/mL penicillin G and 100 μg/mL streptomycin. PK-15 cells were incubated at 37°C with 5% CO_2_.

### Plasmids

pEGFP-ORF5 fusion expression plasmid was constructed in previous study (Lv et al., 2015). DsRed-ORF5 plasmid and pEGFP-LC3 plasmid were constructed in this study. Primer pair ORF5(DsRed) were used to amplify PCV2 ORF5 gene. The DNA product was digested by *Xho*I/*Eco*RI and inserted into similarly digested pDsRed-N1 to generate DsRed-ORF5. Primer pair LC3 were used to amplify LC3 gene from PK-15 cDNA. The DNA product was digested by *Eco*RI/BamH1 inserted into similarly digested pEGFP-N1 to generate pEGFP-LC3. All primers are shown in Table 1. All plasmids were verified by sequencing.

**TABLE 1:** Sequences of primer pairs used for this study.

Gene	Forward primer (5′-3′)	Reverse Primer (5′-3′)	Application
ORF5 (DsRed)	CCGCTCGAGATGTACACGTCATTGTGGGAC	CGGAATTCGGTAGATCATCCCAGGGCG	PCR
LC3	GGAATTCTATGCCCTCAGACCGGCCTTTCA	CGGGATCCTCAGAAGCCGAAGGTTTCCTGGGAG	PCR
ATF4	AGGAGTTCGACTTGGATGCCCTG	AGTGATATCCACTTCACTGCCCAG	qPCR
Cap	CTATCAAGCGAACCACAGTCA	AGACACGGGAAACTTATGATG	PCR
qCap	TCCTCCCGCCATACCATAAC	TGGTCCACATTTCCAGCAGTT	qPCR

### Antibodies

Mouse polyclonal anti-Cap antibody (Abcam, United Kingdom, 1:500), rabbit polyclonal anti-LC3B antibody (Sigma, United States), 1:1000), mouse monoclonal anti-β-actin antibody (NOVUS, United States), 1:1000), rabbit anti-PERK polyclonal antibody (Abcam, United Kingdom, 1:1000), rabbit anti-PERK antibody (phospho T982) polyclonal antibody (Abcam, United Kingdom, 1:1000), rabbit anti-p-eIF2α monoclonal antibody (NOVUS, United States), 1:1000), rabbit anti-eIF2α polyclonal antibody (Enzo Life, United States), 1:1000), rabbit anti-ATF4 polyclonal antibody (Proteintech, United States), 1:1000), rabbit monoclonal antibodies to anti-p-mTOR (Abcam, United Kingdom, 1:1000), and anti-p-AMPK (Abcam, United Kingdom, 1:1000), as well as rabbit polyclonal antibodies to anti-mTOR (Abcam, United Kingdom, 1:1000), anti-p-ERK1/2 and anti-ERK1/2 (CST, United States), 1:1000), and anti-AMPK (CST, United States), 1:1000). HRP (Horseradish peroxidase) Conjugated Goat Anti-Rabbit IgG (H+L) (Abbkine, United States), 1:5000) or Goat anti-Mouse IgG (H+L)-HRP (SUNGENE, China, 1:2000).

### Virus and Reagent

PCV2 and PCV2^ΔORF5^ (ORF5-deficient PCV2 mutant) viruses were constructed for the infectious clone by our laboratory (Lv et al., 2015). PCV2 and PCV2^ΔORF5^ infected PK-15 cells with multiplicity of infection (MOI) of 1 following incubated for 1 h, respectively. Then cells monolayers were rinsed with phosphate buffered saline (PBS, pH = 7.4) to remove unattached viruses and then culture in DMEM medium at 37°C. For pretreating PK-15 cells, autophagy-related agents used in this study were as follows: Rapamycin (Rapa, mTOR inhibitor, MCE, United States), GSK2606414 (PERK inhibitor, MCE, United States), U0126 (MEK inhibitor, MCE, United States), Acadesine (AICAR, AMPK agonist, MCE, United States), Compound C (AMPK inhibitor, MCE, United States), Thapsigargin (TG, Ca^2+^-ATPase inhibitor, Sigma, United States). DMSO was used as a solvent for these chemical reagent as well as a negative control (DMSO < 1%).

### Detection of Cell Viability

To detect cell viability by MTT [3-(4,5-dimethyl-2-thiazolyl)-2,5-diphenyl-2*H*- tetrazolium bromide] assay according to the MTT manufacturer’s instructions. Briefly, PK-15 cells were plated in 96-well plate and incubated at 37°C for 24 h and then added the following agents: Rapa (200 nM), U0126 (20 μM), AICAR (1 mM), Compound C (5 μM), GSK2606414 (1 μM), respectively. PK-15 cells incubated in normal medium supplemented with DMSO solution (DMSO < 1%) used as a vehicle control. Adding 20 μL (pH = 7.4) of MTT solution to each well and continue to incubate for 4 h. Discarding the supernatant and adding 150 μL of DMSO to each well. The optical density was measured at 490 nm using the SpectraMax M2 spectrophotometer (Thermo Fisher Scientific, United States).

### Laser Confocal Microscope

To detect the autophagosomes, PK-15 cells were plated in 10 mm dishes containing of 3 × 10^3^ cells, either infected with PCV2 and PCV2^ΔORF5^ or transfected with DsRed-ORF5, respectively. Those treated cells were incubated at 37°C and 5% CO_2_ for 48 h, 500 μL of 4% paraformaldehyde were added for fixed at 20°C for 20 min, then 500 μL of 1% Triton X-100 were added to permeate at −20°C for 15 min, following blocked with 5% skim milk at 37°C for 2 h. After incubated with rabbit anti-LC3II polyclonal antibody at 4°C for overnight, then FITC-labeled goat anti-rabbit IgG antibody (Abbkine) were added and incubated at 37° for 1 h. DAPI stained for 10 min at room temperature. Each step needs to be washed with PBS for three times. Photos observed and collected under a laser confocal microscope. Experimental procedures followed and modified with Zhu et al. (2012b).

### Western Blotting

In brief, the whole-cell lysates from virus-infected or chemical treated PK-15 cells were resolved by 12% sodium dodecyl sulfate-polyacrylamide gel electrophoresis (SDS-PAGE) and electro-transferred onto the PVDF membrane (Millipore, United States). The membranes were blocked in blocking buffer TBST (pH = 7.4) containing 5% skim milk (BD, United States) at room temperature for 2 h, then incubated with the following antibodies at 4°C for overnight: with antibodies concentration of anti-PCV2 Cap (1:800), anti-LC3II (1:2000), anti-p-PERK (1:1000), anti-PERK (1:800), anti-p-eIF2α (1:500), anti-eIF2α (1:1000), anti-ATF4 (1:800), anti-p-mTOR (1:2000), anti-mTOR (1:1500), anti-p-ERK1/2 (1:1000), anti-ERK1/2 (1:1000), anti-p-AMPK (1:1000) and anti-AMPK (1:2000), respectively, following incubated with HRP conjugated goat anti-rabbit IgG (H+L) (1:20000) or goat anti-mouse IgG (H+L)-HRP (1:5000) antibody for 1 h at room temperature. The PVDF membrane exposure photography using a luminescent liquid and an ECL luminescence imager, target band are gray scaled by Image J-v1.8.0.

### Transmission Electron Microscopy

DsRed plasmid and DsRed-ORF5 recombinant plasmid were used or pretreated with 2 mmol/l 4-PBA (4-phenylbutyrate, an endoplasmic reticulum stress inhibitor) for 2 h, then transfected into PK-15 cells. The cells were taken and prepared as ultrathin sections to be placed under a transmission electron microscope for observation post transfection for 48 h. In briefly, the cells were fixed with 2.5% glutaraldehyde following completely dehydrated by an ethanol dehydration procedure and embedded, sliced with an ultrathin microtome and fixed on the stained plate, placed them on the sample holder after drying. The cells were observed and photographed under a transmission electron microscope applying a voltage of 120 kV at 20 000 magnification.

### Real-Time Quantitative Polymerase Chain Reaction (RT-qPCR)

Briefly, total RNA or DNA was isolated from PK-15 cells at different time points after transfection of the pEGFP-ORF5 plasmid using Trizol reagent (Takara, Japan). Reverse transcribed into cDNA using the FastKing gDNA Dispelling RT SuperMix (TIANGEN, China). The primers were synthesized by AuGCT Biotech (Beijing, China) and sequences are listed in Table 1. PCR parameters used as follows: 95°C for 10 min, 40 cycles at 95°C for 15 s and 60°C for 30 s. To assess the proliferation of PCV2, an standard curve to quantify PCV2 viral DNA was established and the PCV2 viral DNA copies were calculated as described (Lv et al., 2015) and primers are shown in Table 1.

### Statistics

The study data was statistically analyzed using Student *t*-tests. ^∗^*p* < 0.05 and ^∗∗^*p* < 0.01 versus the control group.

## Results

### ORF5 Protein Induces Autophagy

**FIGURE 1:**
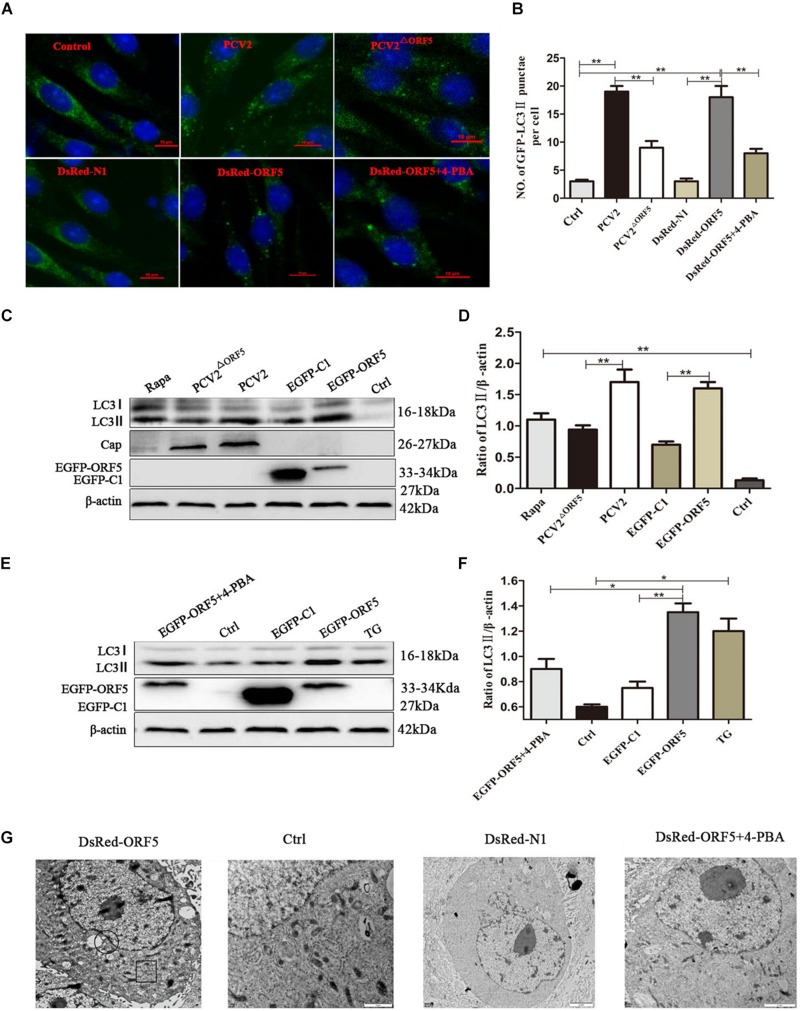
ORF5 induces autophagy in PK-15 cells. **(A)** Formation of autophagosomes, shown as green punctate in PK-15 cells that were analyzed by fluorescence microscopy (scale bar, 10 μm). **(B)** Statistical analysis of panel **(A)**, the number of GFP-LC3 points per cell. **(C)** PCV2 and PCV2^ΔORF5^ were infected with PK-15 cells, and EGFP-ORF5 was transfected into PK-15 cells. All samples were collected 36 h post-infection or post-transfection. **(D)** Perform a *T*-test on the grayscale analysis of the **(C)**, the ratio of LC3 to β-actin. **(E)** PK-15 cells were pretreated with 4-PBA for 2 h and then transfected with EGFP-ORF5. At 36 h post- transfection, immunoblotting was performed. **(F)** Perform a *T*-test on the grayscale analysis of the **(E)**, the ratio of LC3 to β-actin. **(G)** Electron microscopy of autophagosomes and endoplasmic reticulum after transfection of DsRed-ORF5 plasmids. The black box indicates the autophagosome. The scale bar in DsRed-ORF5 panel is 10 μm. In the rest panels is 2 μm. The endoplasmic reticulum structure in the black circle is swollen and rounded. *p < 0.05, **p < 0.01.

To investigate whether the ORF5 protein can elicit autophagy and induce the formation of autophagosomes. The PK-15 cells were transfected with DsRed-ORF5 or EGFP-ORF5, or infected with PCV2 and ORF5-deficient PCV2 mutant strain (PCV2^ΔORF5^), respectively. Autophagy related factors were detected by laser confocal immunofluorescence and Western blotting. The results showed that the number of cells containing spots in LC3 (autophagic vesicles) after PCV2 infection and expression of ORF5 protein was significantly increased than that of PCV2^ΔORF5^ infection group and control group cells (Figures 1A,B). The ratio of LC3-II to β-actin was significantly increased in cells after PCV2 infection and ORF5 overexpression (Figures 1C–F). Rapa and TG were used as positive controls. To further investigate the ability of PCV2 ORF5 in inducing autophagy, 4-PBA (4-phenylbutyrate, an endoplasmic reticulum stress inhibitor) was used to treat ORF5-overexpressed PK-15 cells. The result showed that 4-PBA strongly restored ORF5 induced LC3-II activation (Figure 1E). Next, we employed transmission electron microscopy assay to observe DsRed-ORF5 transfected cells. The result showed multiple autophagosomes can be clearly observed in the cytoplasm of the DsRed-ORF5 group cells while the swelling and rounding of endoplasmic reticulum structure was observed around the nucleus (Figure 1G). However, the presence of autophagosomes in the cytoplasm could not be observed from the cells transfected with DsRed plasmid and 4-PBA pretreatment group (Figure 1G). Taken together, these results demonstrated that PCV2 and ORF5 induce autophagy.

### ORF5 Protein Induces Autophagy via PERK-eIF2α-ATF4 Pathway

**FIGURE 2:**
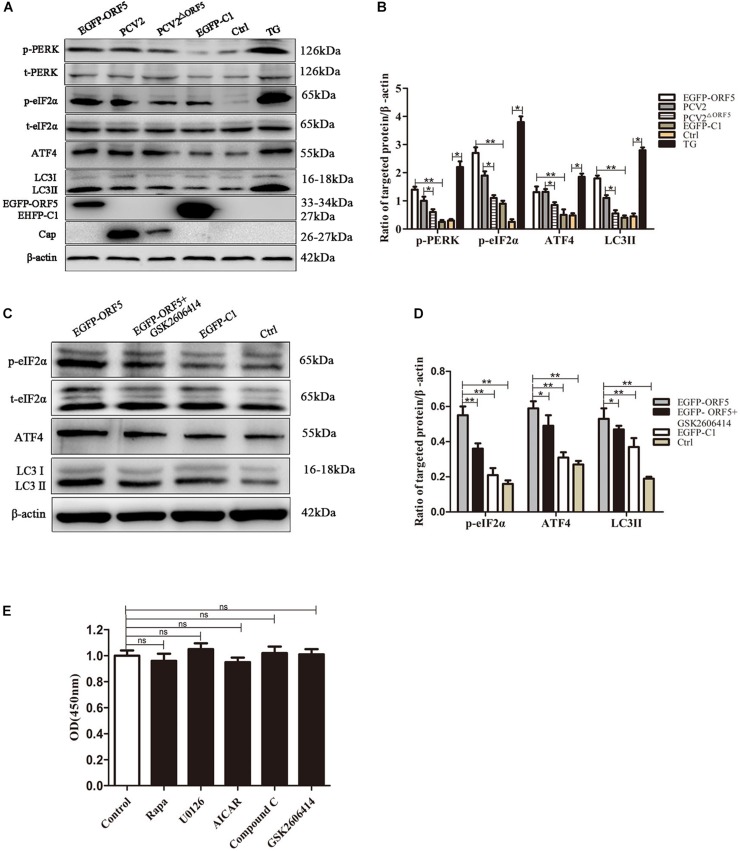
ORF5 protein induces autophagy by PERK-eIF2α-ATF4. **(A)** PK-15 cell transfected with EGFP-ORF5 or infected with PCV2 and PCV2^ΔORF5^ for 36 h. p-PERK, t-PERK, p-eIF2α, t-eIF2α, ATF4, LC3II were detected by immunoblotting. **(B)** *T*-test on the grayscale analysis of the (A), the ratio of targeted protein to β-actin. **(C)** PK-15 cells were treated with GSK2606414 (1 μM), p-eIF2α, t-eIF2α, ATF4, and LC3II were detected by immunoblotting. **(D)** *T*-test on the grayscale analysis of the **(C)**, the ratio of targeted protein to β-actin. **(E)** MTT detects the effect of various chemical treatments on cell viability. All chemical solvents are DMSO (DMSO < 1%). Control only added DMSO (DMSO < 1%) as a negative control. ns, *p* > 0.05 versus the control group. *p < 0.05, **p < 0.01. ns, not significant.

PERK pathway is one of the endoplasmic reticulum pathways that induce the cellular autophagy (Kouroku et al., 2007). After overexpression of ORF5 protein, the expression levels of p-PERK, p-eIF2α, ATF4, and LC3II were detected by immunoblotting. The result demonstrated that p-PERK, p-eIF2α, ATF4, and LC3II were induced by ORF5. Interestingly, the expression of these host factors were also greatly induced by PCV2 infection, but were lees induced by PCV2^ΔORF5^ infection. The protein expression level of p-PERK, p-eIF2α, and LC3II in PCV2-infected group cells was higher than that in PCV2^ΔORF5^ infected cells (Figures 2A,B). This result indicates that ORF5 protein activates the phosphorylation of PERK and eIF2α. To further demonstrate that ORF5 induce autophagy by PERK, the GSK2606414 used to block the PERK pathway and the expression of its downstream factors eIF2α and ATF4 was determined. The results showed that the expression of p-eIF2α and ATF4 were significantly lower in the GSK2606414 pretreatment group than in the EGFP-ORF5 transfection group cells (Figures 2C,D). The MTT assay showed that cell viability was no significant affected among those conditions (Figure 2E). Together, those results proved that ORF5 protein avtivates PERK-eIF2α-ATF4 pathway to induce autophagy in PK-15 cells.

### ORF5 Protein Induces Autophagy by mTOR-ERK1/2-AMPK Pathway

**FIGURE 3:**
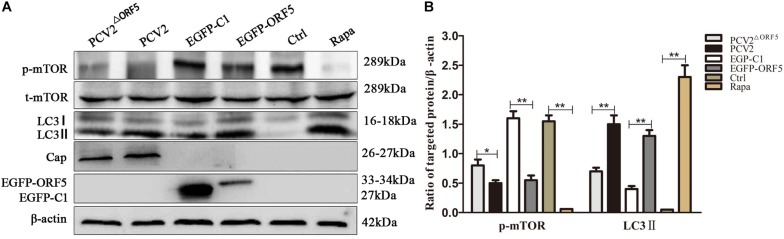
ORF5 protein downregulates phosphorylation of mTOR. **(A)** PK-15 cells were transfected with EGFP-C1 (vector control) or EGFP-ORF5 for 48 h, infected with PCV2 or PCV2^ΔORF5^ for 36 h. p-mTOR, t-mTOR, LC3II were detected by immunoblotting. **(B)** *T*-test on the grayscale analysis of the (A) picture, the ratio of targeted protein to β-actin. *p < 0.05, **p < 0.01.

As shown in Figures 3A,B, the expression of p-mTOR was down-regulated and the expression of LC3 was up-regulated by ORF5 expression in PK-15 cells. Consistently, the p-mTOR was down-regulated to a lower level in WT PCV2 infection group compared to that in the PCV2^ΔORF5^ infection group (Figures 3A,B). In addition, the PCV2 infection up-regulated LC3II expression, while the mutant virus that lacking ORF5 (PCV2^ΔORF5^) partially lost these effects (Figures 3A,B). This result indicates that ORF5 protein inhibited mTOR pathway and promotes cellular autophagy in PCV2 infected cells.

**FIGURE 4:**
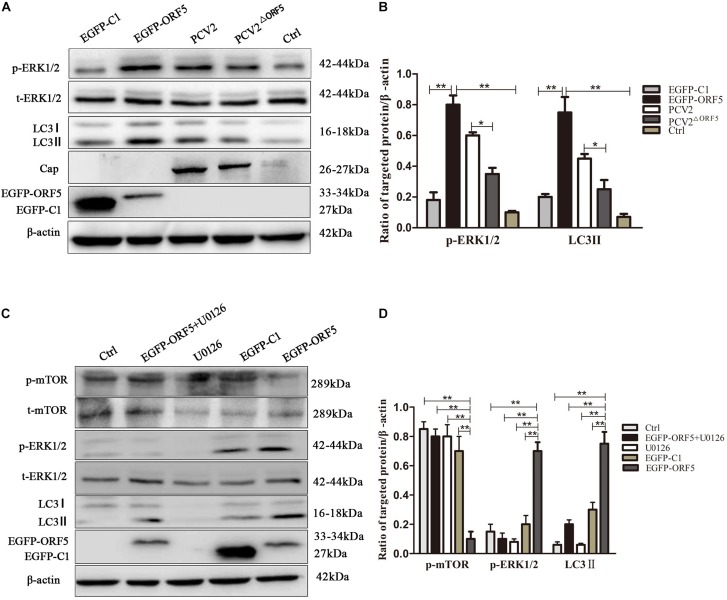
ORF5 protein upregulates phosphorylation of ERK1/2. **(A)** EGFP-ORF5 was transfected for 48 h, PCV2 or PCV2^ΔORF5^ was infected for 36 h, p-ERK1/2, t-ERK1/2, LC3II were detected by immunoblotting. **(B)** Perform a *T*-test on the grayscale analysis of the A picture, the ratio of targeted protein to β-actin. **(C)** PK-15 cells were pretreated with U0126 for 2 h and then transfected with EGFP-ORF5 or EGFP-C1 for 48 h, p-mTOR, t-mTOR, p-ERK1/2, t-ERK1/2, LC3II were detected by immunoblotting. **(D)** Perform a *T*-test on the grayscale analysis of the **(C)**, the ratio of targeted protein to β-actin. *p < 0.05, **p < 0.01.

The phosphorylation level of ERK1/2 and the expression of LC3II protein were significantly up-regulated by ORF5 overexpression in PK-15 cells (Figures 4A,B). Consistently, the p-ERK1/2 and LC3II were induced by PCV2 infection, whereas the expression of p-ERK1/2 and LC3II were less induced by PCV2^ΔORF5^ mutant (Figures 4A,B). The MEK inhibitor U0126 was used to inhibit the ERK1/2 pathway. the results showed that the expression level of p-ERK1/2 and LC3II were down-regulated by MEK inhibitor U0126 (Figures 4C,D). Together, these results indicated that ORF5 protein up-regulated phosphorylation of ERK1/2 in PK-15 cells.

**FIGURE 5:**
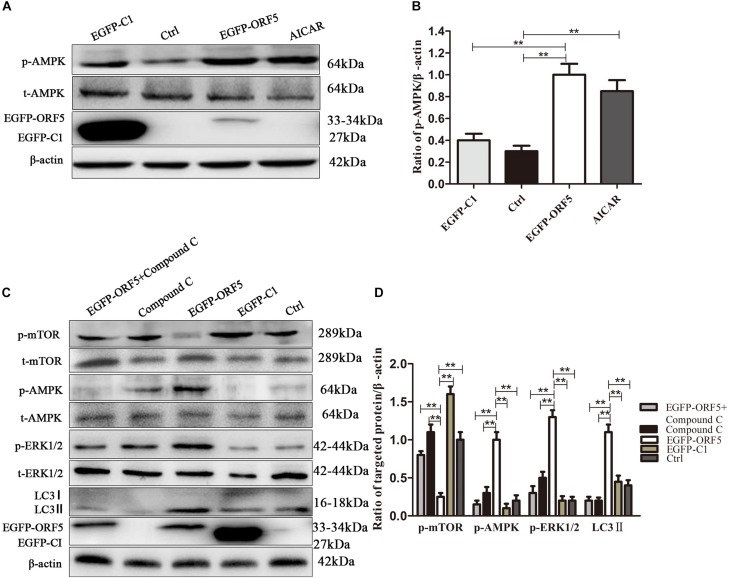
ORF5 protein upregulates phosphorylation of AMPK. **(A)** PK-15 cells were transfected with EGFP-ORF5 or EGFP-C1 for 48 h. AICAR was used as a positive control. p-AMPK and t-AMPK were detected by immunoblotting. **(B)** Perform a *T*-test on the grayscale analysis of the (A), the ratio of targeted protein to β-actin. **(C)** PK-15 cells were pretreated with Compound C for 2 h and then transfected with EGFP-C1, EGFP-ORF5. After 48 h, immunoblot was used to detect p-mTOR, t-mTOR, p-AMPK, t-AMPK, p-ERK1/2, t-ERK1/2, LC3II were detected by immunoblotting 48 h post-transfection. **(D)** *T*-test on the grayscale analysis of the **(C)**, the ratio of targeted protein to β-actin. *p < 0.05, **p < 0.01.

**FIGURE 6:**
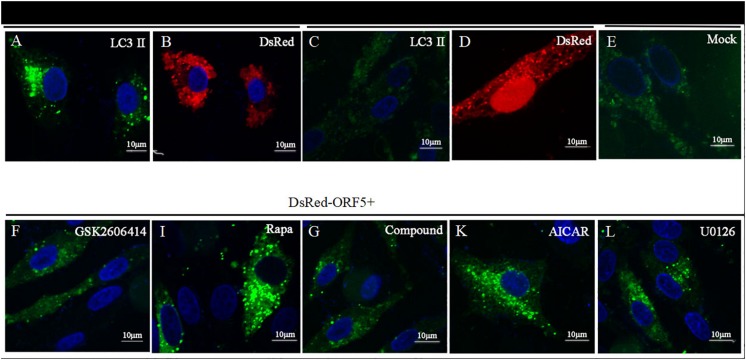
Laser confocal microscope detection of point-like aggregation of LC3. LC3 was detected by laser confocal microscope. **(A)** PK-15 cells were co-transfected with DsRed-ORF5 and EGFP-LC3 for 48 h. **(B)** PK-15 cells were transfected with DsRed-ORF5. **(C)** PK-15 cells co-transfected with DsRed-N1 and EGFP-LC3. **(D)** PK-15 cells were transfected with DsRed-N1. **(E)** Control group. **(F)** PK-15 cells were pretreated with GSK2606414, then co-transfected with DsRed-ORF5 and EGFP-LC3. **(I)** PK-15 cells were pretreated with Rapa, then co-transfected with DsRed-ORF5 and EGFP-LC3. **(G)** PK-15 cells were pretreated with Compound C, then co-transfected with DsRed-ORF5 and EGFP-LC3. **(K)** PK-15 cells were pretreated with AICAR, then co-transfected with DsRed-ORF5 and EGFP-LC3. **(L)** PK-15 cells were pretreated with U0126, then co-transfected with DsRed-ORF5 and EGFP-LC3. ^∗^*p* < 0.05, ^∗∗^*p* < 0.01.

Next, the expression of AMPK pathway was detected in ORF5 expressing cells. The results showed the phosphorylation of AMPK was strongly induced by ORF5, while the AICAR served as a positive control (Figures 5A,B). Blocking the AMPK pathway with a compound C inhibitor significantly reduced p-AMPK, p-ERK1/2 and LC3II expression level in EGFP-ORF5 transfection cells, whereas the p-mTOR is increased (Figures 5C,D). The results of laser confocal showed that the number of dot-like aggregation of LC3 are significantly decreased after inhibiting mTOR-ERK1/2-AMPK pathway (Figure 6). Taken together, we demonstrated that ORF5 protein induced autophagy in PCV2-infected cells via the mTOR-ERK1/2-AMPK pathway.

### ORF5 Protein Promotes Virus Replication

**FIGURE 7:**
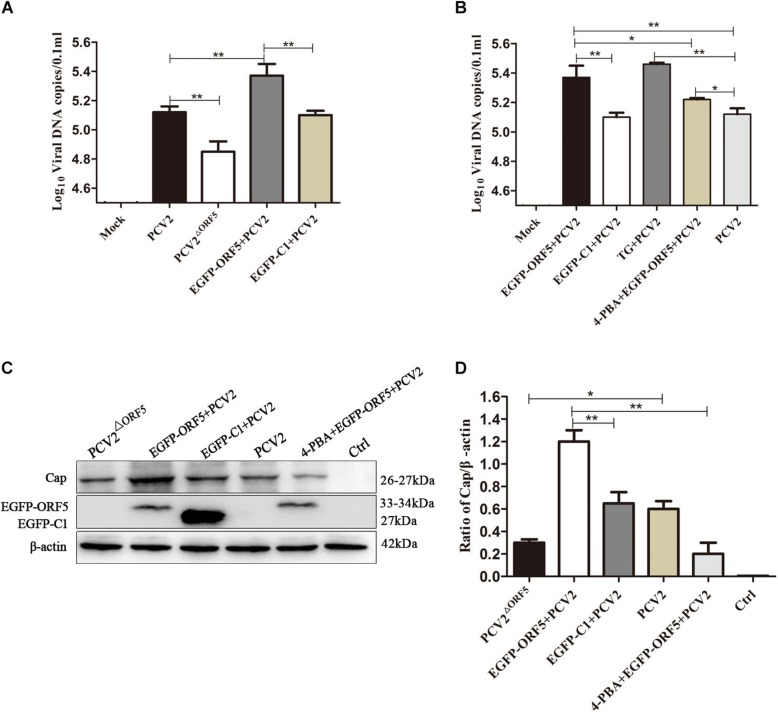
ORF5 protein promotes replication of PCV2. **(A)** PK-15 cells were transfected with EGFP-ORF5 for 12 h and then infected with PCV2 for 36 h. PCV2 DNA copies were quantified by quantitative PCR. **(B)** PK-15 cells were pretreated with TG or 4-PBA for 2 h and then transfected with EGFP-ORF5 or infected with PCV2 for 36 h. DNA copies were quantified by quantitative PCR. **(C)** PK-15 cells were pretreated with 4-PBA for 2 h and then transfected with EGFP-ORF5 for 12 h and then infected with PCV2. At 36 h post-infection, Cap protein and GFP protein were detected by immunoblotting. **(D)** *T*-test on the grayscale analysis of the **(C)**, the ratio of Cap to β-actin. *p < 0.05, **p < 0.01.

By detection of viral genome copy number, we showed the copy number of the PCV2^ΔORF5^ mutant strain was significantly lower compared to PCV2 in infected cells (Figure 7A). Interestingly, the transfection of ORF5 potentiate the replication level of PCV2 (Figure 7A). The inhibition of ERS by 4-PBA significantly decreased viral replication, while the TG pretreatment significantly increased PCV2 level (Figure 7B). Consistently, similar results were obtained by using Western blotting to detect PCV2 Cap expression (Figures 7C,D). Together, the results showed that ORF5 and ORF5-induced autophagy increased the PCV2 replications.

### ORF5 Protein Promotes Replication of PCV2 by PERK-eIF2α-ATF4

**FIGURE 8:**
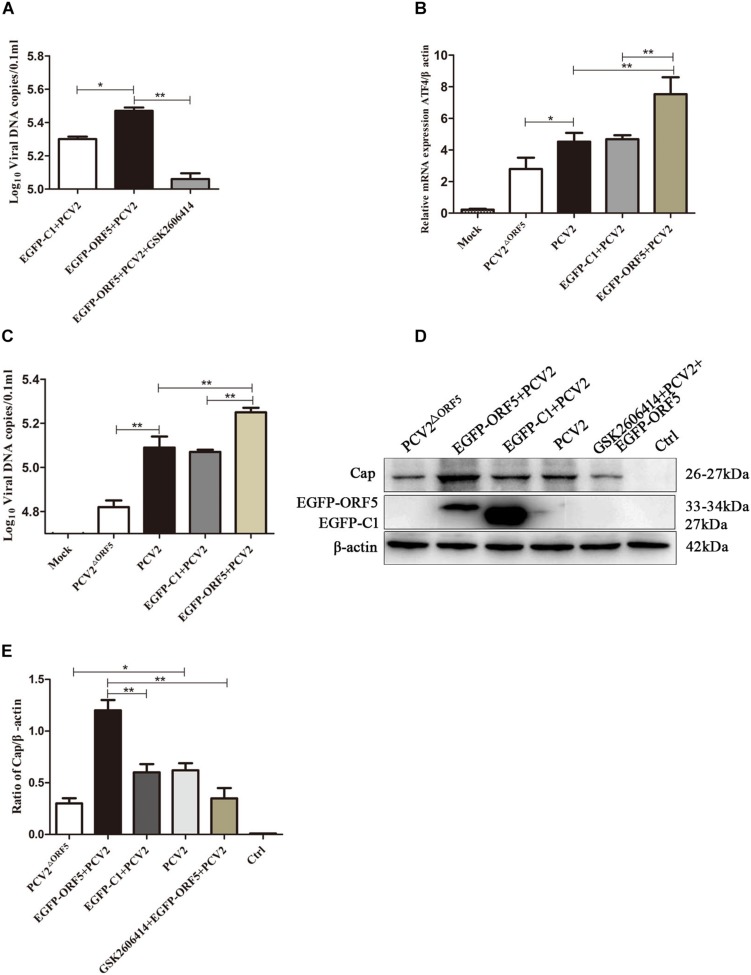
ORF5 protein promotes PCV2 replication by PERK-eIF2α-ATF4 pathway. **(A)** PK-15 cells were pretreated with GSK2606414 for 2 h and then transfected with EGFP-ORF5 for 12 h and infected with PCV2 for 36 h. PCV2 DNA copies were quantified by quantitative PCR. **(B)** PK-15 cells were transfected with EGFP-ORF5 for 12 h and then infected with PCV2 or PCV2^ΔORF5^ for 36 h. The mRNA level of ATF4 was detected by using RT-qPCR. **(C)** PCV2 DNA copies were quantified by quantitative PCR. **(D)** PK-15 cells were pretreated with GSK2606414 for 2 h and then transfected with EGFP-ORF5 for 12 h and then infected with PCV2 or PCV2^ΔORF5^ for 36 h. Cap protein and GFP protein were detected by immunoblotting. **(E)** *T*-test on the grayscale analysis of the **(D)** picture, the ratio of Cap to β-actin. *p < 0.05, **p < 0.01.

The PERK inhibitor GSK2606414 significantly decreased PCV2 replication in ORF5 transfection and PCV2 infected cells (Figure 8A). We further found that the expression of downstream factor ATF4 of the PERK pathway was positively correlated with PCV2 DNA copy number (Figures 8B,C). Western blotting results showed that the PCV2 Cap expression was significantly inhibited by GSK2606414 pretreatment in the EGFP-ORF5 transfection and PCV2 infection group (Figures 8D,E). These results demonstrated that the ORF5 protein increased PCV2 replication and the ORF5 protein promoted the replication of PCV2 via the PERK-eIF2α-ATF4 pathway.

### ORF5 Protein Promotes PCV2 Virus Replication Through mTOR-ERK1/2-AMPK Pathway

**FIGURE 9:**
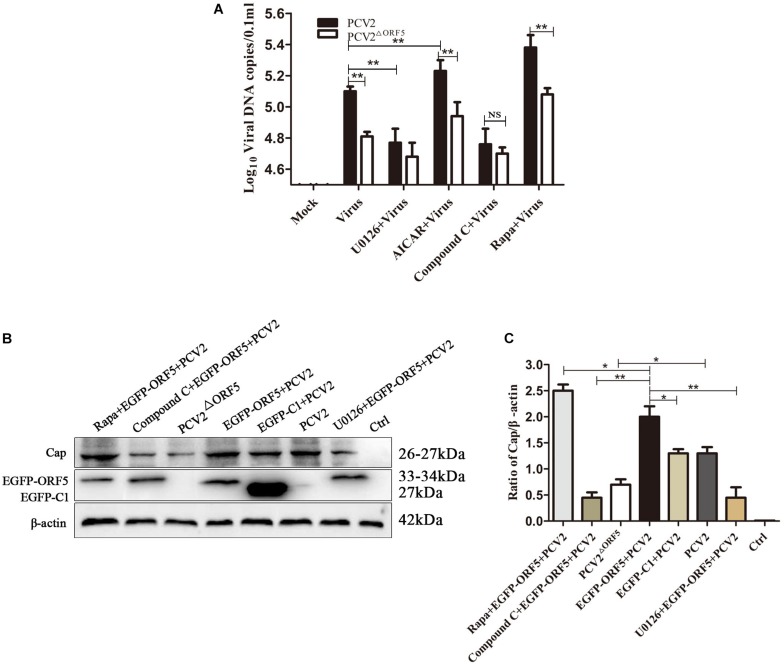
ORF5 protein promotes PCV2 replication via mTOR-ERK1/2-AMPK. **(A)** PK-15 cells were pretreated with U0126, AICAR, Compound C and Rapa for 2 h and then infected with PCV2 or PCV2^ΔORF5^. At 36 h post-infection, PCV2 DNA copies were quantified by quantitative PCR. **(B)** PK-15 cells were pretreated with U0126, AICAR, Compound C and Rapa for 2 h and then infected with PCV2 or PCV2^ΔORF5^. At 36 h post-infection, Cap protein and GFP protein were detected by immunoblotting. **(C)** Perform a *T*-test on the grayscale analysis of the **(B)**, the ratio of Cap to β-actin. *p < 0.05, **p < 0.01.

PK-15 cells infected with PCV2 and PCV2^ΔORF5^ were pretreated with inhibitors or activators of the mTOR pathway, respectively. The results showed that the DNA copy number in Rapa and AICAR pretreatment groups was significantly increased, whereas the treatment of the compound C and U0126 significantly decreased virus replications (Figure 9A). Furthermore, detecting PCV2 Cap expression showed that Rapa pretreatment significantly stimulated Cap expression (Figures 9B,C). The inhibition of AMPK (compound C) and ERK1/2 (U0126) pathways down-regulated Cap expression. These results confirmed that the ORF5 protein induces cellular autophagy through the mTOR-ERK1/2-AMPK pathway to promote PCV2 replication in PK-15 cells.

## Discussion

PCV2 is related to lymphoid degeneration and tissue cell infiltration and induces autophagy (Sinha et al., 2012). The potential function of PCV2 encoding viral proteins has not been fully understood. In the previous study, we demonstrated ORF5 protein of PCV2 is localized to the ER and to induce ERS (Lv et al., 2015). In this study, we focused on the relationship between ORF5 protein and host autophagy. First, we demonstrated that ORF5 protein induced the phosphorylation of PERK and eIF2α. In addition, the expression level of transcription factor ATF4 is also induced by ORF5 protein in PK-15 cells. PERK is one of the sensors that transmits UPR signals in ERS (Ron and Walter, 2007). In following study, we demonstrated that the PCV2 ORF5 protein could also suppress the activation of mTOR pathway, which induces autophagy (Figure 3). It had been reported that Dengue virus (DENV) infection can activate UPR and induce autophagy andPERK-eIF2α and IRE1α-JNK signaling pathways increase autophagy and viral load after DENV infection (Lee et al., 2018). Besides, early infection with dengue virus type 2 (DENV2) triggers PERK-mediated eIF2α phosphorylation (Pena and Harris, 2011). PCV2 infection induces ERS and activates the PERK/eIF2α pathway in UPR, and ERS may play a role in PCV2 infection as part of autophagy and apoptosis (Zhou et al., 2016), as well as the PERK pathway also regulates autophagosome formation (Yang et al., 2017). The Rep and Cap proteins of PCV2 induced UPR by the phosphorylation of PERK with subsequent activation of the eIF2α-ATF4 pathway (Zhou et al., 2017). In the present study, we showed that ORF5 protein activates eIF2α-ATF4 expression by phosphorylating PERK which is consistent with the above studies. We found PCV2 ORF5 phosphorylates PERK and eIF2α also up-regulate the expression of LC3II. This might due to the phosphorylation of PERK accumulates eukaryotic initiation factor 2α (eIF2α), which leads to the formation of Atg5/Atg12/Atg16L complex (Kang et al., 2017; Song et al., 2018). The LC3I is hydrolyzed and cleaved into an active LC3II, thereby mediating the extension of the autophagosome membrane.

The mTOR-ERK1/2-AMPK is also found affected by ORF5 protein in this study. AMPK plays an important role in the regulation of autophagy and proteolysis in mammalian cells (Meley et al., 2006) and autophagy induced by non-starvation-related factors act primarily through activation of AMPK (Hoyer-Hansen et al., 2007). Autophagy mediates enhanced AMPK activity in human breast and cervical cancer cells (Hoyer-Hansen et al., 2007) and activated AMPK initiate autophagosome formation by activating PIK3C3/VPS34 complex or by inhibiting mTOR (Xie et al., 2016) and regulate ERK1/2 (Kim et al., 2012). These opposite effects are due to complex intermodulation and constraints among the AMPK, ERK1/2, and mTOR signaling pathways. ORF5 induces phosphorylation of AMPK, thereby enhancing the activity of ERK1/2, but negatively regulating mTOR since inhibiting the phosphorylation of mTOR, the cleavage of LC3-I to LC3-II is enhanced, and the autophagy is induced.

Some viruses can promote their own replication and maturation by converting the Atg5/Atg12/Atg16L complex to LC3, which is the major component of autophagy (Doring et al., 2018). Autophagosomes promotes virus replication, such as hepatitis B virus (HBV) (Li et al., 2011), adenovirus (Rodriguez-Rocha et al., 2011) and encephalomyocarditis virus (Zhang et al., 2011). Some RNA viruses replicate in the cytoplasm can apply the bilayer membrane structure of autophagic vacuoles as a site of attachment to resist cell clearance. For example, severe acute respiratory syndrome virus (Coronavirus, SARS-CoV) and mouse hepatitis virus (MHV) can simultaneously detect LC3 II and virus in autophagosomes after infection of cells (de Haan et al., 2010). In conjunction with that PCV2 promotes its own replication by autophagy, ORF5 protein phosphorylates the PERK-eIF2α pathway and activates the expression of the downstream factor ATF4, which significantly increases the viral capsid protein as well as the PCV2 DNA copy number, the replication capacity of PCV2 is reduced when PERK was blocked by GSK2606414. This indicates that ERS produced by ORF5 promotes the formation of autophagosomes and has a beneficial effect on the replication of PCV2. This consistent with previous studies that PCV2 infection of PK-15 cells induces autophagy and significantly decreases PCV2 virus titer after interfering with autophagy (Zhu et al., 2012a, b). Again, it demonstrated that ORF5 protein affects PCV2 replication through the PERK pathway. Phosphorylation of AMPK is reduced while phosphorylation of mTOR is induced when ERK1/2 blocked, resulting in the DNA copy number of PCV2 decreased and inhibiting DNA replication. On the contrary, phosphorylation of ERK1/2 and AMPK promotes replication of PCV2 after inhibition of mTOR by Rapa, it confirmed that the ORF5 protein enhances its own replication by inducing autophagy.

Although we have studied the relationship between ORF5 protein, autophagy and viral replication, there are still some issues need further investigation. Even though PCV2 infection can activate both TSC2 and ERK1/2 (Zhu et al., 2012a), the relationship between ERK1/2 and TSC2 and the interaction between ORF5 protein and TSC2 still remains unknown. Herpes simplex virus type 1 protein ICP34.5 inhibits autophagy by interfering with eIF2α kinase signaling, and directly bind to beclin1 in primary neurons (Talloczy et al., 2002). HBV activates autophagy by modulating beclin1, simultaneously activating PI3K/Akt/mTOR and adenosine monophosphates-activated protein kinase (AMPK) (Xie et al., 2018). These studies indicate Beclin1 is also one of the key proteins in the autophagy regulatory pathway. Thus, the role of beclin1 in the regulation of autophagy induced by PCV2 ORF5 protein needs further explorations.

## Conclusion

In summary, in this study, we demonstrated that PCV2 ORF5 induces autophagy in PK-15 cells. Subsequently, we revealed that the PCV2 ORF5 protein play essential roles in PCV2-induced PERK-eIF2α-ATF4 pathway activations and suppressed mTOR phosphorylation. Finally, we convincingly showed ORF5 promotes PCV2 replication level via PERK-eIF2α-ATF4 and mTOR-ERK1/2-AMPK pathways.

## Data Availability Statement

All datasets generated for this study are included in the article/supplementary material.

## Author Contributions

KG, JL, and YZ designed the experiments. JL and YJ carried out the experiments, collected the data, wrote this manuscript, and checked and revised the manuscript. QF, ZF, YS, PX, YH, and YF helped to finish the experiments. XZ, XX, and YZ contributed the materials. JL and YS contributed the data analysis.

## Conflict of Interest

The authors declare that the research was conducted in the absence of any commercial or financial relationships that could be construed as a potential conflict of interest.
